# Effect of heterozygous β-thalassaemia trait on coronary atherosclerosis via coronary artery disease risk factors: a preliminary study

**Published:** 2007-07

**Authors:** M Hashemi, E Shirzadi, Z Talaei, L Moghadas, I Shaygannia, M Yavari, N Amiri, H Taheri, H Montazeri, H Shamsolkottabi

**Affiliations:** Noor Hospital, Isfahan, Iran; Medical University of Isfahan, Isfahan, Iran; Medical University of Isfahan, Isfahan, Iran; Medical University of Isfahan, Isfahan, Iran; Medical University of Isfahan, Isfahan, Iran; Medical University of Isfahan, Isfahan, Iran; Medical University of Isfahan, Isfahan, Iran; Medical University of Isfahan, Isfahan, Iran; Medical University of Isfahan, Isfahan, Iran; Sina Hospital, Isfahan, Iran

## Abstract

**Background:**

Thalassaemia is considered the most common genetic disorder worldwide. An association between the heterozygous β-thalassaemia trait and myocardial infarction has previously been observed. However, the relationship between heterozygous β-thalassaemia and atherosclerosis, considering other coronary artery disease (CAD) risk factors, has remained unclear.

**Methods:**

A case-control study was conducted to evaluate the hypothesis that thalassaemia minor affects the likelihood of atherosclerotic plaque formation. Blood counts and blood chemistry data as well as traditional risk factors from 1 363 patients referred to heart centres for coronary angiography were recorded. Heterozygous β-thalassaemia was diagnosed by the presence of hypochoromic-microcytic anaemia, ferritin levels > 12 ng/ml and haemoglobin-A2 levels > 3.5.

**Results:**

Chi-squared analysis showed that the prevalence of heterozygous β-thalassaemia was not significantly different between patients with and without CAD (*p* > 0.05). Multivariate logistic regression analysis using CAD as the dependent variable and traditional risk factors, haematocrit, ferritin levels and heterozygous β-thalassaemia as independent variables, did not show any significant difference either. Independent two-tailed student’s *t*-tests showed that haematocrit levels were statistically different (*p* = 0.000) between CAD^+^ and CAD^−^ groups, but low-density lipids (LDL), high-density lipids (HDL), triglycerides (TG), total cholesterol and serum ferritin levels were not statistically different (*p* > 0.05).

**Conclusion:**

The prevalence of heterozygous β-thalassaemia in the case group was not significantly different from the control group. This case-control study did not support the hypothesis that thalassaemia minor affects the likelihood of atherosclerotic plaque formation.

## Summary

Thalassaemia is considered the most common genetic disorder worldwide.^1^,^2^ In line with the controversy over the relationship between coronary artery disease (CAD) and the heterozygous β-thalassaemia trait (Hβ-TT) over the past decades, a lower incidence of acute myocardial infarction has recently been reported in thalassaemia carriers.^3^,^4^,^5^ However, this protective effect was observed only in males.^6^,^7^

A reduction in risk factors, particularly serum cholesterol levels,^8^,^9^ lower blood viscosity due to decreased haematocrit and haemoglobin levels,^10^ and a lower incidence of arterial hypertension^6^,^7^ has been proposed to be responsible for the protective role of Hβ-TT. On the other hand, a decrease in haemoglobin levels in anaemia has been reported as an independent risk factor for cardiovascular disease.^11^,^12^ Severe iron overload in patients with β-thalassaemia^13^-^18^ may actually be a risk factor for atherosclerosis. 19-23 An increase in PF3 activity in thalassaemic patients due to abnormal erythrocytes leads to activation of the coagulation mechanisms.^24^ Also some studies suggest that impaired glucose tolerance and hypertriglyceridaemia is more prevalent in β-thalassaemia.^25^,^26^

Since these findings are so controversial, we studied the association between coronary atherosclerosis and Hβ-TT. Lipid profiles, haematocrit and serum ferritin levels were also measured to understand the role of these factors in the relationship.

## Materials and methods

The target population in this case-control study was 1 363 patients referred for coronary angiography to heart centres in Shahid Chamran and Sina Hospitals, Isfahan, Iran [631 patients with angiographically approved coronary artery disease (CAD^+^) and 608 patients without coronary artery disease (CAD^−^)]. Some of the patients were invited to participate in this study by telephone or letter. Informed consent was obtained from the patients before enrolling them in the study. The study protocol was approved by the ethical committee of the University of Isfahan.

Demographic and baseline data consisting of age, gender and drug history (antihypertensive, antidiabetic and antilipid drugs) were recorded for each patient. Height and weight were measured and body mass index was calculated (kg/m^2^). Cigarette smoking was based on the patient’s self-report and it was calculated as pack/year; patients who had ceased smoking for more than three years were considered non-smokers.^27^

Blood was collected in two tubes. A plain tube was used for enzymatic determination of glucose, cholesterol, triglycerides (TG), low-density lipoprotein (LDL) and high-density lipoprotein (HDL) by technical Ra1000 auto-analyzer. A second tube containing EDTA as anticoagulant was used for complete blood count (CBC) analysis [including red blood cells (RBC), haemoglobin, haematocrit, mean corpuscular volume (MCV), mean corpuscular haemoglobin (MCH) and mean corpuscular haemoglobin concentrations], using the auto-counter AC920EO (SWELAB).^28^

For patients with MCV less than 78 fl and MCH less than 27 pg/cell, haemoglobin electrophoresis on acetate cellulose gel at pH 8.6 was run.^29^ For this group of patients, haemoglobin-A2 was quantitatively determined by ion-exchange choromatographic spectrophotometry, and serum ferritin levels were determined by RIA method.^30^,^31^ Hβ-TT was diagnosed by the presence of hypochoromic-microcytic anaemia (MCV < 78 fl and MCH < 27 pg/cell), ferritin levels above 12 ng/ml and haemoglobin-A2 levels above 3.5.^29^

During coronary angiography, systolic and diastolic blood pressures were recorded invasively. All the angiographic CDs were analysed at the angiographic laboratory by two experienced cardiologists who were blinded to the clinical and biological data. Coronary atherosclerosis was defined as the presence of any atheroma (identified as luminal irregularity) according to coronary angiography, by visual assessment in each vessel.

Chi-squared analysis was performed to assess the association between CAD and Hβ-TT. In addition, stepwise multivariate logistic regression analysis (SPSS 11.5 software) was used to determine the mechanism by which Hβ-TT potentially affects coronary atherosclerosis. A *p*-value < 0.05 was considered as statistically significant.

## Results

A total of 1 363 patients were enrolled in the study; 608 subjects (41.6%) were angiographically without any atheroma plaque (CAD^−^), and 883 (60.4%) were male. The average age was 56.23 ± 10.97 (range: 20−94 years). Chi-squared analysis showed that the prevalence of Hβ-TT was not significantly different between patients with and without CAD (*p* > 0.05). Data are shown in Table 1.

**Table 1:** The Prevalence Of Thalassaemia In Patients With And Without CAD

*CAD*		*Heterozygous β-thalassaemia trait*		
		*Positive*	*Negative*	*Total*
Negative	Male	7	276	283
	Female	8	313	321
	Total	15	589	604
Positive	Male	12	444	456
	Female	4	155	159
	Total	16	599	615

Stepwise multivariate logistic regression analysis was performed using CAD as a dependent variable and other factors such as age, gender, BMI, smoking, blood pressure, LDL, HDL, TG, total cholesterol, fasting blood glucose, haematocrit and ferritin levels and heterozygous β-thalassaemia as independent variables. Results did not show any significant differences. Means and standard deviations of haematocrit, LDL, HDL, TG, total cholesterol and serum ferritin levels are shown in Table 2.

**Table 2:** Comparison Of TG, HDL, LDL, Total Chlosterol, Haematocrit And Serum Ferritin Level Between Two Groups. Data Are Presented As Mean ± SD

	*Thalassaemia (1)*	*Thalassaemia (2)*	p*-value*
Triglyceride	196.225 ± 120.472	213.540 ± 144.755	NS
HDL	46.096 ± 14.251	44.365 ± 12.113	NS
LDL	100.109 ± 39.751	105.805 ± 40.407	NS
Total cholesterol	209.064 ± 60.681	211.145 ± 49.016	NS
Haematocrit	36.154 ± 4.123	42.997 ± 5.227	S
Ferritin	110.747 ± 166.130	60.600 ± 82.627	NS

NS: not significant, S: significant

Independent two-tailed student’s *t*-tests showed that haematocrit was significantly different (*p* = 0.000) but LDL, HDL, TG, total cholesterol and serum ferritin levels were not significantly different between thalassaemic patients and the normal group (*p* > 0.05).

## Discussion

In our study, despite Hβ-TT being more prevalent in the control than the case group, there was no statistically significant relationship between coronary artery disease and Hβ-TT. From previous controversial studies, we would have expected a protective effect of β-thalassaemia due to low haemoglobin, haematocrit and lipoprotein levels.^8^-^10^,^32^-^35^ While Sarnake *et al.* showed anaemia as an independent risk factor for cardiovascular disease,^11^ Shahriari *et al.* considered that low haemoglobin in thalassaemia might protect thalassaemic patients from ischaemic heart disease.^36^

Some studies have demonstrated Hβ-TT as a protective factor for CAD due to the following possible mechanisms:^37^-^40^ (1) a reduction in risk factors, particularly with regard to serum cholesterol and LDL;^8^,^9^,^32^,^33^ (2) lower blood viscosity due to decreased haematocrit and haemoglobin;^7^,^10^ and (3) lower incidence of hypertension in thalassaemia carriers.^6^,^7^ Although they have confirmed an inverse relationship between Hβ-TT and the risk of MI, the relationship between Hβ-TT and atherosclerotic plaque has not been well characterised.

Furthermore other articles considered β-thalassaemia as a factor that may increase the risk of CAD and plaque formation by other mechanisms such as: (1) existence of iron overload in β-thalassaemia, which may actually be a risk factor for atherosclerosis;^13^-^20^,^22^,^23^ (2) an increase in PF_3_ activity in thalassaemic patients, which leads to increased coagulation;^24^ and (3) an increase in impaired glucose tolerance and triglycerides.^25^,^26^ However, this concept remains controversial.

It was beyond the scope of this article to analyse this controversy in detail. In our case-control study we compared the prevalence of Hβ-TT in 1 363 patients who had undergone angiography (their angiography report was either CAD^+^ or CAD^−^). Statistical analysis (chi-square) for each group revealed no statically significant difference between patients with and without β-thalassaemia. A separate analysis for men and women again showed no difference.

We had recorded demographic and baseline data as well as blood pressure, BMI, cigarette smoking, TG, LDL, HDL, total cholesterol, haematocrit, fasting blood glucose and serum ferritin levels. Stepwise multivariate logistic regression analysis was performed using CAD as the dependent variable and LDL, HDL, TG, total cholesterol, fasting blood glucose, haematocrit, ferritin and Hβ-TT as independent factors. Results did not show any significant differences; even the *p*-value was increased. Independent two-tailed student *t*-analysis demonstrated significant difference between haematocrit levels in thalassaemic patients versus the normal group, but no significant difference was observed in serum ferritin levels between these two groups.

Although our study was not randomised, we believe the use of a control group that had no atherosclerotic plaque with angiography was valid for testing the hypothesis that Hβ-TT has no effect on coronary atherosclerosis, which was contrary to other previous studies. This contradiction may have been due to different indicators of CAD being used in our study from others. While they founded their results on clinical features such as MI, we considered atherosclerotic plaque detected in angiography to be a more sensitive indicator.

There were some limitations to this study. First, the study included only subjects who were referred for coronary angiography to our heart centres. Second, the low prevalence of Hβ-TT in our sampled population, in comparison with what was expected from previous studies necessitates more patients to raise the power of statistical tests. We suggest further studies with a larger sample size to confirm our findings.
